# Sport Practice, Fluid Reasoning, and Soft Skills in 10- to 18-Year-Olds

**DOI:** 10.3389/fnhum.2022.857412

**Published:** 2022-03-14

**Authors:** Tommaso Feraco, Chiara Meneghetti

**Affiliations:** Department of General Psychology, University of Padova, Padua, Italy

**Keywords:** sport practice, soft skills, physical activity, cognitive abilities, fluid reasoning

## Abstract

Engaging in physical activity and sports has been associated with various cognitive abilities and other personal characteristics. The contemporary link between doing sports and personal attributes such as soft skills and an individual’s cognitive abilities have yet to be investigated, however. This study aims to analyze the association between years of practicing a sport, cognitive abilities (in terms of fluid reasoning), and personal attributes (in terms of soft skills). A large sample of 1,115 individuals (10–18 years old) completed the Cattell test (measuring fluid reasoning) and answered a questionnaire measuring six soft skills (adaptability, curiosity, initiative, leadership, perseverance, and social awareness). A multivariate regression analysis show that, after controlling for age and gender, participants’ years of practicing a sport were positively associated with three soft skills (i.e., initiative, leadership, and perseverance) and with fluid reasoning. No differences emerged between team and individual sport practitioners. Our findings suggest an association between practicing sports, which entails more than just physical activity, and both cognitive abilities (fluid reasoning) and other important personal characteristics, such as soft skills.

## Introduction

Physical activity and sports are fundamentally important in late childhood and adolescence, their benefits affecting various aspects of an individual’s life, and their mental and physical health. That is why international organizations support them and recommend that people aim for (or maintain) adequate and healthy levels of physical activity and engage in sports (World Health Organization., 2019). Researchers are also paying more and more attention to how engaging in physical activity and sports is associated with other domains, such as academic performance (St Clair-Thompson and Gathercole, 2006), or with positive aging (Salas-Gomez et al., 2020). For older children and adolescents, doing sports may also have other important benefits. It has been found related to cognitive abilities (perception, attention, visuospatial abilities, intelligence; Voss et al., 2010; Voyer and Jansen, 2017; Scharfen and Memmert, 2019) and individual characteristics such as personality and soft skills (Zaff et al., 2003; Khasanzyanova, 2017; de Prada Creo et al., 2020; Feraco et al., 2022). The latter association might be particularly important at a malleable age, such as adolescence, when an individual’s cognition and personality take shape (Paus, 2005; Steinberg, 2005; Heckman, 2011; Thompson et al., 2019). Results of studies sustaining the hypothesis of a correlation between sports or physical activity and cognitive abilities or soft skills are mixed (Carson et al., 2016; Li et al., 2017; Salas-Gomez et al., 2020), however, making it difficult to precisely estimate the strength of this association.

Cognitive abilities [which include the abilities involved in mentally handling information (Carroll, 1993)], and soft skills [or the personal qualities that positively regulate emotions, thoughts, and goal-directed behaviors (Park et al., 2004; Robles, 2012)] are fundamentally important to an individual’s wellbeing and success in adulthood (McClelland, 1973; Sternberg, 1997; Bertua et al., 2005; Strenze, 2007; Heckman and Kautz, 2012; Bruna et al., 2019). They are also essential in adolescence, as school students rely on these skills for their academic achievement (Lounsbury et al., 2009; Roth et al., 2015; MacCann et al., 2020; Feraco et al., 2021b). Importantly, cognitive abilities and soft skills are thought to be malleable, as suggested by specific interventions (Durlak et al., 2011; Jaeggi et al., 2011; Shipstead et al., 2012; Hodzic et al., 2018; Schutte and Malouff, 2019). Identifying which practical and ecological activities correlate with better cognitive abilities and soft skills could consequently be hugely important, and sports might be a good candidate (Gomez-Pinilla and Hillman, 2013; Carson et al., 2016; Voyer and Jansen, 2017; Bidzan-Bluma and Lipowska, 2018; Hernández-Mendo et al., 2019).

Previous studies found that expert practitioners of various sports had stronger cognitive abilities, in terms of their visuospatial abilities, attention, processing speed, executive functions, or general cognitive abilities (Voss et al., 2010; Moreau et al., 2011; Heppe et al., 2016; Scharfen and Memmert, 2019; Feraco et al., 2021a; Meneghetti et al., 2021). Meta-analyses examining the link between sports and cognitive abilities found only small-to-medium effect sizes, however, and noted small sample sizes, multiple testing approaches, and a low statistical power as major shortcomings of most of the research conducted in this field (Voss et al., 2010; Voyer and Jansen, 2017; Scharfen and Memmert, 2019). Such limitations may lead to the magnitude of the effects being exaggerated (Button et al., 2013; Gelman and Carlin, 2014). Any effect would also presumably be even smaller in populations of non-elite or non-expert sports practitioners, like the majority of adolescents who engage in sports. Our first aim here is therefore to examine the association between sports and cognitive abilities in a large group of preadolescents and adolescents after calculating the sample size needed to detect small effect sizes (*r* = 0.15; Scharfen and Memmert, 2019). We focus on fluid reasoning as a valid proxy for general cognitive abilities (the *g* factor), as it has been shown to correlate similarly with the various subcomponents of the *g* factor during adolescence (Breit et al., 2019).

Then, to add to the literature on the beneficial effects of sports, a second aim is to test the association between a structured and continuous engagement in a sport and the sphere of soft skills. This set of malleable, positive characteristics should influence an individual’s wellbeing and success in life by regulating their thoughts, behaviors, and emotions (Robles, 2012; Feraco et al., 2021b). For the purposes of the present study, we consider the six soft skills included in the World Economic Forum model (World Economic Forum., 2016) because of their importance to wellbeing, education, and job success: adaptability, curiosity, leadership, initiative, perseverance, and social awareness. Despite the attention being paid to soft skills around the world (Cinque, 2016; European Commission, 2016; Ministry of Education University and Research [MIUR], 2018; World Economic Forum, 2020), research on the link between sports and soft skills is scarce, and warrants specific studies. It is important to establish whether such an association exists, and whether it is worth promoting sports in adolescents as a way to sustain their soft skills. The few studies conducted to date support the hypothesis that people practicing sports or other extracurricular activities report better soft skills (Zaff et al., 2003; Holt et al., 2013; Arat et al., 2014; Khasanzyanova, 2017; Mızrak et al., 2017; de Prada Creo et al., 2020; Feraco et al., 2022). Practicing a sport is not just a matter of physical and motor abilities. It also demands that people continuously face challenges relating to many difficult situations and interpersonal relationships, and work on their identity and personal qualities to succeed in what they are doing (Eccles, 1999; Clark et al., 2015; Fakhretdinova et al., 2020). Practicing a sport has been found associated with students’ leadership (Holt et al., 2013; Clark et al., 2015; de Prada Creo et al., 2020), perseverance (Fourie and Potgieter, 2001; Guillén and Laborde, 2014), and emotional intelligence (Laborde et al., 2017), but also with other soft skills, such as initiative, adaptability, and curiosity (Feraco et al., 2021b,^2022^).

To sum up, the aim of the present study is to investigate the cross-sectional association between years of practicing a sport and both cognitive abilities (in terms of fluid reasoning) and personal characteristics (in terms of soft skills) in a large sample of 10- to 18-year-olds. This age group was chosen because both cognitive abilities and personality are malleable at this time of life (Steinberg, 2005; Heckman, 2011), and because few studies have tested these hypotheses in adolescents. We hypothesize that years of practicing a sport should correlate positively: with fluid reasoning, given that expert sportspeople perform better than non-experts in various cognitive tasks (Voss et al., 2010; Voyer and Jansen, 2017); and with soft skills because practicing a sport also involves a host of relational and personal competences (Guillén and Laborde, 2014; de Prada Creo et al., 2020; Feraco et al., 2021b). We examine whether all six soft skills considered, or some of them in particular (such as leading a team or persevere toward one’s aim for long time, will be related) are associated with the practice of a sport. Both types of association (with cognitive abilities and with soft skills) are expected to be small, given the findings of previous meta-analyses and the fact that we analyze yearly increments (Scharfen and Memmert, 2019; Feraco et al., 2021b).

## Materials and Methods

### Participants

The study sample consisted of 1,115 individuals (521 males, *M*_age_ = 13.51, SD_age_ = 2.16) from 10 to 18 years old (see Table 1 for the sample’s characteristics), who were enrolled on a voluntary basis. Of these individuals, 984 engaged in amateur sports for at least a year (*M*_year_ = 5.71, SD = 3.68), and the other 131 had never engaged in any sport. The amount of practice was measured in terms of the number of years respondents had engaged in sport during their lives, rated as: 0; 1–2 years; 3–4 years; 5–6 years; 7–8 years; 9–10 years; 11–12 years; or 13–14 years. Respondents also indicated how many hours a week they spent practicing their sport (see Table 1), and 436 of them also specified the type of sport they were practicing at the time of data collection.

**TABLE 1:** Characteristics of the study sample.

Age	10	11	12	13	14	15	16	17	18
Males	43	74	105	76	71	45	53	43	11
Females	38	59	113	89	73	80	70	58	14
Sport practitioners	76	122	187	148	133	110	107	82	19
Total	81	133	218	165	144	125	123	101	25
Hours per week	4.64 (2.77)	4.01 (2.58)	4.14 (3.11)	4.55 (2.99)	5.26 (3.2)	4.64 (3.36)	4.68 (3.16)	4.38 (3.56)	3.76 (3.13)

*Number of participants, females, males, sports practitioners (who had engaged in a sport for at least a year), and hours of practice per week, with means and standard deviations (in brackets).*

The sample size needed was calculated using a power analysis. We simulated 10,000 datasets for different sample sizes based on a theoretical covariance matrix in which a small association (*r* = 0.15; Scharfen and Memmert, 2019) between years of sport practice and the seven dependent variables was hypothesized. On each dataset, we ran the analyses described in the section “Results,” and calculated how many times all hypothesized associations were contemporary significant (*p* < 0.05). It emerged that 1100 participants sufficed to obtain a power of 0.99.

### Materials

All the scales used in the study showed acceptable reliability coefficients, as calculated on the actual sample (0.64 < α < 0.79).

#### Soft Skills

The soft skills questionnaire (Feraco et al., 2021b) measures the six soft skills included in the personal qualities branch of the World Economic Forum. (2016):

*Adaptability*, or the ability to adapt positively to new and uncertain situations in everyday life (e.g., “I’m scared by situations that are new to me.”; Martin et al., 2012);

*Curiosity*, or the epistemic desire to acquire new knowledge (e.g., “Whenever I see something new, I try to understand what it is.”; Berlyne, 1960);

*Initiative*, or deliberate personal growth referred to general everyday life situations (e.g., “If a decision has to be made, I make it.”; Robitschek et al., 2012):

*Leadership*, or the characteristics typical of leadership, such as being the reference person in a group, or supporting and motivating others (e.g., “I can take the lead in team efforts.”; Peterson and Seligman, 2004);

*Perseverance*, or the general tendency to work hard to reach aims despite difficulties (e.g., “Faced with a difficult situation, I don’t give up.”; Duckworth et al., 2007);

*Social awareness*, or sense of responsibility for the community and the environment (e.g., “It’s important that all people be treated equally.”; Peterson and Seligman, 2004).

Each subscale is composed of six items (except for leadership, with four items) scored on a 6-point Likert scale. Each total is derived from the sum of its corresponding items.

#### Cognitive Abilities

*Culture-Free Intelligence Test* (Cattell, 1940). This test measures fluid reasoning with four different time-limited tasks that involve: (i) finding the image that completes a sequence (12 items, 3 min); (ii) finding the image that differs from the others (14 items, 4 min); (iii) finding the image that completes a matrix (12 items, 3 min); and (iv) finding the image that presents the same spatial relationships as a target figure (8 items, 2.5 min). A total score was calculated from the sum of the items correctly answered in all four tasks.

### Procedure

We collected data in two phases (520 participants responded between January and March 2019; another 595 responded between January and February 2020, before the COVID-19 pandemic spread). Participants were recruited through schools. In September 2019 and September 2020, we contacted the principals of numerous schools in northern and central Italy. After obtaining their agreement, consent forms were distributed to the parents of potential participants. After receiving the parents’ consent, we organized our data collection. Eighteen-year-old participants completed their own consent form.

A trained psychologist collected the data during school time and under the supervision of a class teacher. Participants first completed a personal information section, indicating their age, gender, and engagement in sports. Then they answered the soft skills questionnaire and performed the Cattell test. The order of presentation of the two measures was randomized between classes. For the questionnaire, participants were told there were no right or wrong answers. For each of the four cognitive tasks, they read the instructions and answered the sample items together with the experimenter, who also told them about the time limit, and stopped them when their time was up. The procedure took less than 1 h to complete in each class.

## Results

All analyses were run using the “lavaan” package in R (Merkle and Rosseel, 2018; R core team., 2020). A multivariate regression analysis was used to study the effect of practicing a sport (years) on the levels of soft skills (adaptability, curiosity, leadership, perseverance, and social awareness) and cognitive abilities (fluid reasoning). The dependent variables (soft skills and fluid reasoning), but not the years of sport, were scaled (*M* = 0; SD = 1) to make the results comparable and easier to interpret. Age and gender were always added as covariates to control for their effect on the dependent variables (Gur et al., 2012; Voyer et al., 2017; Heintz et al., 2019).

**FIGURE 1:**
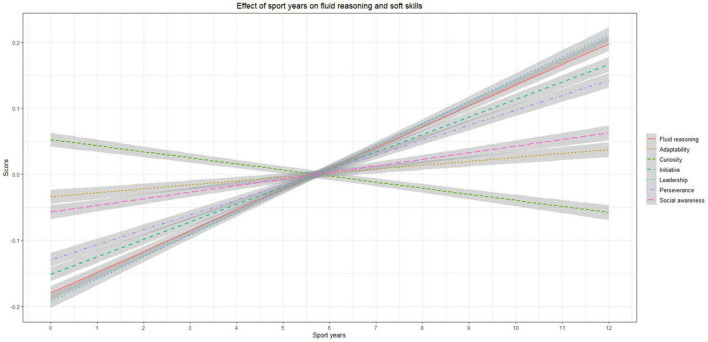
Association between number of years of practicing a sport, soft skills, and fluid reasoning. 15% confidence intervals are shown for readability. Soft skills and fluid reasoning are scaled to mean = 0; SD = 1.

The results of the multivariate regression model show that the years of practicing a sport correlated with four of the seven dependent variables considered, after accounting for the effect of age and gender (see Figure 1). We found that the number of years spent practicing a sport correlated positively with initiative (*p* < 0.001; β = 0.06), leadership (*p* < 0.001; β = 0.08), perseverance (*p* < 0.001; β = 0.06), and fluid reasoning (*p* < 0.01; β = 0.04), but not with adaptability (*p* > 0.05; β = –0.00), curiosity (*p* > 0.05; β = –0.01), or social awareness (*p* > 0.05; β = 0.02), with beta estimates indicating the amount of standardized increase for every 2 years. Descriptively, as concerns the covariates: age was positively associated with fluid reasoning, and negatively associated with all the soft skills except adaptability and social awareness, which remained stable with age; gender differences only emerged for adaptability (in favor of males), and social awareness (in favor of females). See Table 2 for the complete results including the covariates.

**TABLE 2:** Results of multivariate regression analysis.

Dependent variable	Predictor	*B*	*SE*	Z	CI [2.5, 97.5]
Fluid reasoning	Age	0.14***	0.01	1.21	(0.11;0.16)
	Females	0.06	0.06	1.10	(-0.05;0.17)
	Years of practicing sports	0.04**	0.02	2.58	(0.01;0.07)
Adaptability	Age	-0.00	0.01	-0.10	(-0.03;0.03)
	Females	-0.22***	0.06	-3.61	(-0.33; -0.10)
	Years of practicing sports	0.01	0.02	0.74	(-0.02;0.04)
Curiosity	Age	-0.04***	0.01	-3.22	(-0.07; -0.02)
	Females	0.03	0.06	0.53	(-0.09;0.15)
	Years of practicing sports	-0.01	0.02	-0.67	(-0.04;0.02)
Initiative	Age	-0.06***	0.01	-4.48	(-0.09; -0.04)
	Females	0.10	0.06	1.66	(-0.02;0.21)
	Years of practicing sports	0.06***	0.02	3.91	(0.03;0.10)
Leadership	Age	-0.05***	0.01	-3.44	(-0.07; -0.02)
	Females	-0.00	0.06	-0.07	(-0.12;0.11)
	Years of practicing sports	0.07***	0.02	4.64	(0.04;0.11)
Perseverance	Age	-0.08***	0.01	-5.93	(-0.11; -0.06)
	Females	0.09	0.06	1.60	(-0.02;0.21)
	Years of practicing sports	0.06***	0.02	3.66	(0.03;0.09)
Social awareness	Age	-0.02	0.01	-1.62	(-0.05;0.00)
	Females	0.46***	0.06	7.82	(0.34;0.57)
	Years of practicing sports	0.02	0.02	1.49	(-0.01;0.06)

Dependent variables (fluid reasoning and soft skills) are scaled to mean = 0; SD = 1. **p < 0.01 and ***p < 0.001. SE = standard error; z = test statistic; β = beta coefficient; CI = confidence interval; Females indicates the difference between males and females, with males as the baseline.

We also checked whether practicing different sports might affect the dependent variables differently by running a second multivariate linear regression model with the type of sport as the predictor, and age and gender as covariates. This was done after dichotomizing the types of sport as team sports (e.g., basketball, handball) and individual sports (e.g., tennis, athletics) (Laborde et al., 2016). Only data for the subsample of participants who provided information about the sports they engaged in and those who reported never engaging in any sport were considered (*N* = 567). The analysis yielded no significant results regarding the type of sport (*p* > 0.05; β ≤ | 0.05|).

## Discussion

International organizations promote the value of physical activity and sports in the general population (World Health Organization., 2019) because they are good for our physical and mental health, but the literature suggests that they may have other benefits. Practicing sports may also influence our cognitive abilities (e.g., visuospatial skills, attention, perception), and various aspects of our personality or character, such as soft skills (Eccles, 1999), particularly during childhood and adolescence (Paus, 2005; Steinberg, 2005; Heckman, 2011; Thompson et al., 2019). Hence our present effort to further analyze how practicing sports (in terms of the number of years involved) correlates with cognitive abilities and soft skills in 10- to 18-year-olds (an age when these abilities and skills are still malleable). A large sample (1,115 participants) was examined to test the presumably small (as suggested by Scharfen and Memmert, 2019) effects of years of practicing a sport on seven dependent variables (cognitive abilities, adaptability, curiosity, initiative, leadership, perseverance, and social awareness).

The results of our multivariate regression analysis confirmed our hypotheses regarding cognitive abilities and three soft skills, which correlated significantly with the number of years spent practicing a sport. As expected, these correlations were small (ranging from 0.04 for cognitive abilities to 0.07 for leadership), which goes to show the importance of large sample sizes to detect these associations (Voss et al., 2010; Voyer and Jansen, 2017; Scharfen and Memmert, 2019). They nonetheless support the existence of a positive link between years of practicing a sport and important personal characteristics (cognitive abilities and soft skills). Our results also suggest that every additional year of practice counts: for children engaging in sports from early on, at 8 years old, their cognitive abilities would potentially have a 0.20 standardized benefit after 10 years. This is far from negligible, considering that: cognitive abilities are important throughout our lives (Ree et al., 1994; Roth et al., 2015); these sports are usually practiced freely, not as an activity intended to train cognitive abilities; and sports have enormous benefits on other aspect of a child’s life (e.g., physical and mental health; World Health Organization., 2019).

The effects of sport on soft skills seems particularly interesting. Our sample of adolescents seemed to identify improvements in their cognitive abilities as they grew older, but a decline in their soft skills (the association between participants’ age and soft skills was constantly negative and significant). This might be a problem, given the importance of soft skills in their future lives (Heckman and Kautz, 2012; World Economic Forum, 2020). However, we found that perseverance, leadership, and initiative correlated with years of practicing a sport, in line with previous reports (Fourie and Potgieter, 2001; Holt et al., 2013; Guillén and Laborde, 2014; Clark et al., 2015; de Prada Creo et al., 2020; Feraco et al., 2021b). This might be due to the specific demands of sporting activities. For instance, perseverance might be a core characteristic of sportspeople because it is rare for anyone to see results immediately after a single training session. Learning a new technique or movement might initially be frustrating, and it can only be mastered by staying focused, continuing to practice, and coping with setbacks. At the same time, sports involve competitions that can often last a whole year with unexpected results, and failures need to be adequately managed, avoiding the temptation to give up, in order to achieve good results at the end of the year – and this takes perseverance. People engaging in sports must also constantly take responsibility for their actions and make decisions all the time they are playing, as they know it will affect their own or their team’s results. They have to learn to take action that is appropriate and well-timed, so practicing a sport could really empower an individual’s personal initiative. There is also a clear association between practicing a sport and developing leadership (de Prada Creo et al., 2020). Whether they engage in individual or team sports, practitioners almost never work by and for themselves. Every action they take has consequences on their own performance, and that of others (teammates, trainers, sponsors), and they must nurture their ability to collaborate with others (planning training sessions, understanding and respecting the role of every member of the group) in order to reach the goals they have set themselves.

In short, our findings support the claim that practicing sports can nurture people’s cognitive abilities and personality (Eccles, 1999). It can strengthen an individual’s sense of identity and achievement (Eccles, 1999; Clark et al., 2015). These added values of practicing sports deserve to be better investigated in experimental or longitudinal studies. Some soft skills - adaptability, curiosity, and social awareness - did not reveal any significant associations with years of practicing sports in our sample of adolescents, but further research might be able to shed more light on their role. Importantly, we also found no difference between individuals practicing team versus individual sports in the seven dependent variables considered here: specific research might better investigate this issue.

While the above considerations seem plausible, they are only the fruit of speculation because we adopted a cross-sectional approach that prevents us from drawing any conclusions on the causality of the effects identified. It may be, for instance, that more perseverant people keep practicing sports. A longitudinal approach would be better suited to investigating any improvements in a given individual’s cognitive abilities and soft skills. We only administered one test on fluid reasoning as a measure of cognitive abilities, disregarding many other abilities that might be influenced by practicing a sport (e.g., processing speed, perception, attention, visuospatial abilities; Voss et al., 2010; Voyer and Jansen, 2017; Scharfen and Memmert, 2019). We also limited the soft skills considered to six, though there are many others that it would be worth testing. It might be rightly argued, too, that we only considered the number of years our participants had spent practicing a sport, without considering the amount of time they dedicated to it (e.g., hours per week) or the level of expertise they had reached, which might also affect the results (Meneghetti et al., 2021).

## Conclusion

We analyzed the association between sports, in terms of years of practicing a sport, and seven variables: cognitive abilities (fluid reasoning), and six soft skills. Our findings support the conviction that practicing a sport not only promotes physical and mental health in general but may also be associated with important cognitive abilities (fluid reasoning) and personal characteristics (soft skills). Even if the effects identified were small, preadolescents and adolescents who had been practicing a sport for more years scored higher in terms of their cognitive abilities and three soft skills, i.e., initiative, leadership, and perseverance.

## Data Availability Statement

The datasets presented in this study can be found in online repositories. The names of the repository/repositories and accession number(s) can be found below: doi: 10.6084/m9.figshare.17429738.

## Ethics Statement

The studies involving human participants were reviewed and approved by the University of Padua’s Ethics Committee for Research in Psychology. Written informed consent to participate in this study was provided by the participants’ legal guardian/next of kin.

## Author Contributions

TF and CM contributed to the conception and design of the study, wrote sections of the manuscript, revised the manuscript, and read and approved the submitted version. TF performed the statistical analysis, organized the database, and wrote the first draft of the manuscript.

## Conflict of Interest

The authors declare that the research was conducted in the absence of any commercial or financial relationships that could be construed as a potential conflict of interest.

## Publisher’s Note

All claims expressed in this article are solely those of the authors and do not necessarily represent those of their affiliated organizations, or those of the publisher, the editors and the reviewers. Any product that may be evaluated in this article, or claim that may be made by its manufacturer, is not guaranteed or endorsed by the publisher.
